# Two-Photon Imaging for Non-Invasive Corneal Examination

**DOI:** 10.3390/s22249699

**Published:** 2022-12-11

**Authors:** Ana Batista, Pedro Guimarães, José Paulo Domingues, Maria João Quadrado, António Miguel Morgado

**Affiliations:** 1 Coimbra Institute for Biomedical Imaging and Translational Research (CIBIT), University of Coimbra, 3000-548 Coimbra, Portugal; 2 Institute for Nuclear Sciences Applied to Health (ICNAS), University of Coimbra, 3000-548 Coimbra, Portugal; 3 Department of Physics, Faculty of Science and Technology, University of Coimbra, 3004-516 Coimbra, Portugal; 4 Department of Ophthalmology, Centro Hospitalar e Universitário de Coimbra, 3004-561 Coimbra, Portugal; 5 Coimbra Institute for Clinical and Biomedical Research (iCBR), Faculty of Medicine, University of Coimbra, 3000-548 Coimbra, Portugal

**Keywords:** cornea, two-photon imaging, fluorescence lifetime imaging, second-harmonic generation, corneal collagen organization, optical metabolic imaging

## Abstract

Two-photon imaging (TPI) microscopy, namely, two-photon excited fluorescence (TPEF), fluorescence lifetime imaging (FLIM), and second-harmonic generation (SHG) modalities, has emerged in the past years as a powerful tool for the examination of biological tissues. These modalities rely on different contrast mechanisms and are often used simultaneously to provide complementary information on morphology, metabolism, and structural properties of the imaged tissue. The cornea, being a transparent tissue, rich in collagen and with several cellular layers, is well-suited to be imaged by TPI microscopy. In this review, we discuss the physical principles behind TPI as well as its instrumentation. We also provide an overview of the current advances in TPI instrumentation and image analysis. We describe how TPI can be leveraged to retrieve unique information on the cornea and to complement the information provided by current clinical devices. The present state of corneal TPI is outlined. Finally, we discuss the obstacles that must be overcome and offer perspectives and outlooks to make clinical TPI of the human cornea a reality.

## 1. Introduction

Over the past decades, our understanding of corneal anatomy and physiology in health and disease has greatly improved due to the introduction and continuous improvement of corneal imaging methodologies. Clinical modalities, such as laser scanning confocal microscopy (LSCM) and anterior segment optical coherence tomography (AS-OCT), have enhanced our knowledge of the pathophysiological mechanisms and improved our ability to diagnose corneal diseases. Nevertheless, current clinical modalities are still limited. LSCM relies on changes in the tissue refraction index as contrast mechanism. Therefore, functional imaging is unattainable. The major limitations of AS-OCT are its low transverse resolution (order of several tens of micrometers) as well as its inability to assess the cell’s metabolic state [1,2]. None of these imaging modalities can assess the collagen organization within the corneal stroma.

Two-photon imaging (TPI) of the cornea has shown great potential for corneal evaluation and it can become a major asset in clinical practice. Being a multimodal approach, TPI overcomes the limitations of the current clinical tools. It enables combined structural and functional examination of the tissue, as well as assessment of the stroma structural organization. Thus, a complete assessment of the corneal status is within reach using TPI.

This article reviews the current state of TPI for corneal examination. It includes a brief overview of corneal anatomy and morphology and of the principles of two-photon imaging, including a more detailed presentation of the physics of Second Harmonic Generation (SHG). The endogenous sources of contrast for corneal TPI are presented. Methodological topics discussed include the methods for quantification of tissue organization, based on SHG signal, instrumentation for TPI, including recent advances in laser sources and photodetectors, safety considerations for in vivo human eye irradiation, and data and image analysis methods. Finally, the article reviews published studies on TPI of the cornea and its clinical applications.

## 2. Cornea Anatomy and Morphology

**Figure 1:**
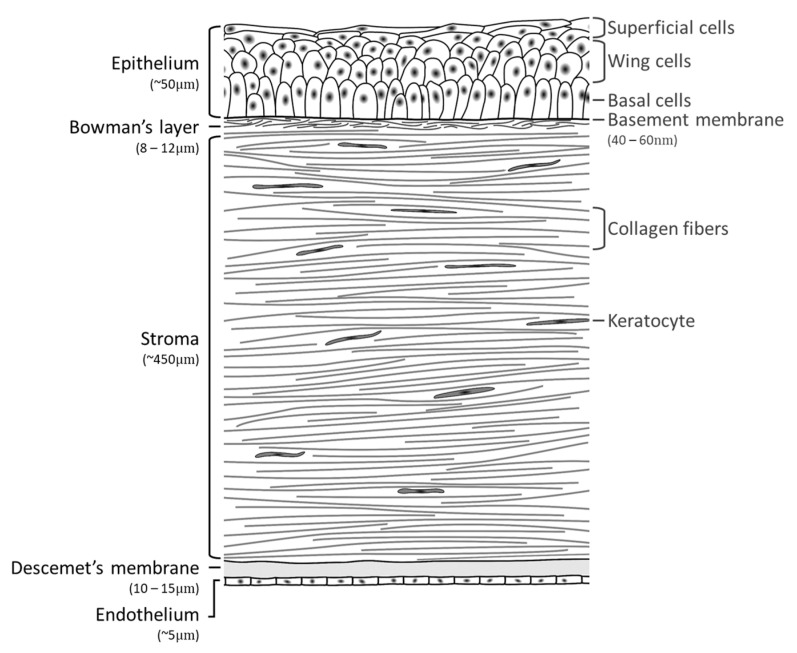
Representation of the human cornea cross-section. Reproduced from [7].

The human cornea (Figure 1) is the transparent and avascular tissue in the outermost anterior part of the eye [3,4,5,6]. Its primary function is light transmission and refraction. It also plays a role in biodefense by preventing the entrance of foreign organisms [5].

The human cornea thickness increases gradually towards the periphery from 500 μm up to 700 μm thick. It is composed of five main layers: the epithelium, Bowman’s layer, stroma, Descemet’s membrane, and endothelium. Optically, the cornea is an asymmetric biconvex lens, with an average refractive power of 43 diopters (2/3 of the total refractive power of the eye) [8].

Corneal transparency, essential for light transmission, is assured by the specific structural organization of the stromal collagen fibers, the absence of blood vessels, the absence of myelin sheath in the corneal nerves, and the ability to maintain a controlled hydration state.

### 2.1. Epithelium

The corneal epithelium is composed of 5 to 7 layers of squamous nonkeratinized stratified cells (8 to 10 in the corneal periphery) with a total thickness of 50–56 μm. The innermost layer is composed of a single column of basal cells, with the remaining layers containing intermediate wing cells and superficial cells, held together by tight junctions. The epithelium has several functions, namely, an important role in optical transparency, physical protection by providing a barrier to fluid and microorganisms’ penetration, and tear film stabilization.

The epithelium is maintained by a continuous process comprising cell production, proliferation with differentiation and migration to the epithelial surface, and loss by shedding. This process requires a peripheral influx of cells derived from stem cells in the limbal basal epithelium and Palisades of Vogt. The basal cells are the only epithelial cells that proliferate, differentiating into wing cells and, later, into superficial cells, within a period of 7–14 days [5]. It is the epithelial tissue with the fastest turnover rate, which enables quick recovery after injury [3].

The superficial cells are organized in 2–3 layers, presenting different maturation stages, with most exterior cells undergoing continuous shedding. The wing cells are organized in 2–3 layers centrally and 4–5 in the periphery. The basal cells are germinative cells, organized in a single layer anchored on the basal membrane of the epithelium. This acellular membrane is composed of collagen, mainly type IV, laminin, and proteoglycans. It participates, together with the basal cells hemidesmosomes, in the adhesion of the epithelial cells to the stroma, through anchoring fibrils that penetrate in the stromal structure [9].

The epithelium also contains non-epithelial cells, namely lymphocytes, melanocytes, macrophages, and Langerhans cells.

### 2.2. Bowman’s Layer

The Bowman’s layer is an acellular layer 10–15 μm thick, decreasing from the center to the periphery. Its function is to maintain the epithelial organization and integrity, and to separate the epithelium from the stroma. It is present only in the cornea of primates [5].

The Bowman’s layer corresponds to an organization of type I and III collagen fibers and proteoglycans. In the deepest regions, the collagen fibers increase their diameter and transform gradually into stromal collagen fibers.

In the past, much importance was given to the Bowman’s layer for maintaining corneal transparency [10]. Today, it is known that this transparency remains after Bowman’s layer excision in photorefractive keratectomy. The same happens with epithelial adhesion. In the absence of the Bowman’s layer, the regeneration of the epithelium occurs upon the stroma, and there are studies indicating that the normal epithelial maturation is not affected [10,11,12].

### 2.3. Corneal Stroma

The corneal stroma is a connective tissue composed of keratocytes, collagen type I lamellae, and extracellular matrix, found around and between collagen fibers and keratocytes [13]. The central thickness of the corneal stroma varies between 500 and 540 μm, being higher in the periphery where it can reach 700 μm. Thus, the stroma contributes to about 90% of the total corneal thickness [5].

Keratocytes are specialized fibroblasts and correspond to 3–5% of the corneal stroma. Its turnover is about 3 years [5]. These cells maintain the structure of the lamellae and extracellular matrix by synthesizing proteoglycans and collagen. In the face of aggression, the quiescent keratocytes migrate to restore the injured structures, turning into myofibroblasts. Myofibroblasts produce the extracellular matrix, collagen degradation enzymes, metalloproteinases, and cytokines that contribute to tissue repair. Their contraction ability also contributes to the healing process [5]. In excimer laser surgeries, keratocytes increase their activity in areas close to the intervention, a condition that can last for several months [5].

The collagen present in the stroma is mainly type I. Nonetheless, other fiber-forming collagen types have been found in the cornea: II, III, and V [14,15]. Stromal collagen is highly organized into layers, named lamellae, composed of parallel collagen fibers, also called fibrils. Their diameters and interfibrillar distances are very uniform in the central cornea, a feature that is essential for maintaining corneal transparency. The fibers have a diameter of about 31 nm in diameter [16,17], with a quasi-uniform distance between them of about 42–51 nm [5,18]. These fibers are parallel to each other in each lamella and range from limbo to limbo. There are around 200 lamellae in the stroma, lying parallel and interweaved, with oblique orientation between them. Their thickness can reach up to 2 μm and their width may reach 0.2 mm [17].

The organization of the collagen lamellae changes along corneal depth. In the anterior and mid-stroma, the lamellae are highly interwoven, while in the posterior stroma, the lamellae become thicker and less interweaved, presenting a more regular arrangement parallel to the surface of the cornea [17,19]. At the periphery, in the interface between stroma and the sclera, the lamellae become disorganized, and the tissue loses its transparency.

The interfibrillar space contains proteoglycans [5] that attach to collagen fibers in an orderly manner, which is essential for interfibrillar ordering and spacing and, therefore, to maintain corneal transparency. Currently, it is known that an arrangement of the collagen fibers in a perfect hexagonal lattice with the interfibrillar spacing being a fraction of the wavelength of the light, as proposed by Maurice [20], is not a requirement for achieving corneal transparency. It is sufficient that the fibers’ diameter is uniform and the distance between adjacent fibers is constrained [17,21]. This last requirement is achieved by a balance between repulsive and attractive interactions, with the proteoglycans taking part in both. On the one hand, the negatively charged proteoglycan coating around the fibers attracts positive charged ions. These, in turn attract water molecules by osmosis, which will exert pressure on the fibers. On the other hand, proteoglycan bridges connect neighbor fibers that pull the connected fibers to each other [17]. The resulting fibers arrangement guarantees that constructive interference of the waves scattered by the collagen fibers occurs only in the forward direction.

### 2.4. Descemet’s Membrane

The Descemet’s membrane is a thin layer (8–12 μm) that remains lightly attached to the stroma. It corresponds to the basal membrane of the endothelium and is produced by endothelial cells. Its thickness increases from about 3 μm at birth, at a rate of about 1 μm per decade [22]. Observation by electron microscopy allows us to distinguish two regions: anterior and posterior. The anterior region, about 2 μm thick, is formed by thin fibrils of type VIII collagen with a hexagonal arrangement. The increase in the thickness of the Descemet’s membrane throughout life occurs primarily in the posterior region, which consists of collagen type IV and VIII, fibronectin, and glycoproteins [23]. Due to this constitution, it presents great elasticity and resistance, even greater than that of the stroma, in the face of traumatic and inflammatory aggressions.

When damaged, the Descemet’s membrane easily detaches from the stroma and regenerates rapidly. However, in a situation of rupture, and once it is covered by endothelial cells, the membrane does not regenerate. Endothelial cells when stimulated by inflammation, trauma, or genetic disorders can lead to an increase in collagen deformation, causing a thickening of Descemet’s membrane.

### 2.5. Endothelium

The corneal endothelium corresponds to a monolayer of contiguous cuboid cells, which form a typical hexagonal mosaic. The thickness of this layer is 4 to 6 μm. Endothelial cells have a high metabolic activity.

The endothelial cells do not present specialized adhesions to the Descemet’s membrane. However, interdigitations with various types of junctional complexes are present between them. Intercellular spacing is very reduced but allows the penetration of small molecules from the anterior chamber. Those interdigitations facilitate the increase in cell size that compensates for constant cell loss [24]. In fact, the endothelium does not have the capacity to renew itself. This leads to a loss in the population of endothelial cells with age. When an endothelial cell is damaged, the neighbor cells increase in size, thus resulting in a structural change called polymegathism. Endothelial loss is also manifested by pleomorphism, associated with an increase in the permeability of these cells [24]. Under the age of five, the endothelial cell density is about 3500 cells/mm^2^. The critical value below which the endothelial function is at risk is around 800 cells/mm^2^.

The most important function of the endothelium is to control the penetration of substances from the aqueous humor, to ensure normal hydration of the stroma, fundamental for maintaining corneal transparency. Endothelial cells have an ion transport system that opposes the entry of aqueous humor into the stroma. An osmotic gradient of Na^+^ is present between the aqueous humor and the stroma. This gradient results in Na+ influx from aqueous humor to stroma and K^+^ transport in the opposite direction. This activity is called endothelial pump. Na^+^, K^+^-ATPase located on the cell membrane provides the most important activity for this action. Despite the alterations produced by age, the barrier and pump effect of the endothelium do not change.

### 2.6. Corneal Nerves

The cornea is one of the most innervated tissues in the human body. This enervation is achieved by the ophthalmic nerve, a division of the trigeminal nerve, via the anterior ciliary nerves and those surrounding the conjunctiva. The nerves penetrate the cornea in a central and anterior direction, in a radial fashion, originating the branches that innervate the mid and anterior stroma. Most branches divide into smaller branches that penetrate superiorly into the epithelium [25]. At the interface between Bowman’s layer and the anterior stroma, they organize into the sub-basal nerve plexus. Then, they cross the epithelium, constituting the epithelial network [25,26], and terminate at the level of the superficial cell layer [27].

The density of epithelial nerves is higher at the central cornea than at the periphery. The density of epithelial nerves in the normal human adult cornea, expressed as a percentage of the area, is reported as 18.78 ± 2.06 at the central area and 11.05 ± 2.27 at the periphery [26]. At the stromal level, the density of nerve trunks is about 32.6 ± 6.4, and a progressive reduction in corneal nerve density with age is reported [26].

Corneal nerves do not have a myelin sheath [28] and are surrounded by Schwann cells [29]. Nevertheless, Muller et al. showed that nerves located at the peripheral anterior stroma can be myelinated [30].

It is possible to non-invasively image the corneal nerves through corneal confocal microscopy. These images have been used to evaluate diabetic peripheral neuropathy. A comprehensive review on this subject can be found in [31].

## 3. Principles of Two-Photon Imaging

The basis of TPI is the near simultaneous, nonlinear interaction of two photons with molecules. From their interaction, two distinct processes can occur depending on the molecule in question: (1) absorption of photon energy followed by relaxation via fluorescence emission (TPEF); (2) induction of polarization of the second order in the electric field leading to second-harmonic generation (SHG). These imaging modalities can be easily combined into a single TPI platform. Having specific contrast mechanisms, TPEF and SHG can be used concomitantly to provide a complete analysis of the tissue. In this section, we provide an overview of the theoretical principles of TPEF and SHG.

### 3.1. Two-Photon Excited Fluorescence

The simultaneous interaction of two photons with the molecules was initially theorized by Dr. Maria Göppert-Mayer in 1931 [32], and first demonstrated experimentally by Kaiser and Garrett in 1961, after the introduction of laser sources [33]. The first TPEF microscope for biological samples was introduced by Denk, Strickler, and Webb in 1990 [34].

**Figure 2:**
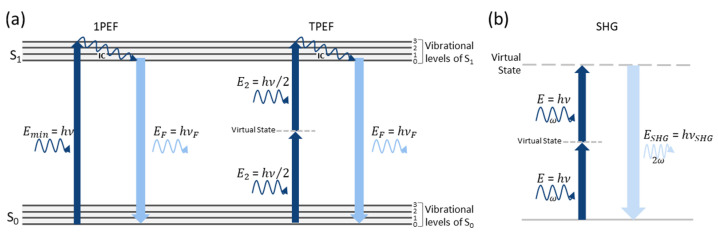
Jablonski diagrams of one-photon excitation (1PEF) and two-photon excitation (TPEF) fluorescence (**a**) and energy level diagram of second-harmonic generation (SHG) (**b**). The photon energy absorption by the molecule promotes its excitation from the ground state ($S_{0})$ to a higher energy state ($S_{1}$), followed by internal conversion (IC) and relaxation by photon emission. In SHG, the energy of two photons with angular frequency ω is converted into a single photon with double the energy and angular frequency 2ω. E—energy; $E_{min}$—energy difference between $S_{0}$ and $S_{1}$; $E_{2}$—half of $E_{min}$; $E_{F}$—energy of fluorescence photons; $E_{SHG}$—energy of SHG photons; h—Planck’s constant; ν—frequency; $\nu_{F}$—frequency of fluorescence photons; $\nu_{SHG}$—frequency of SHG photons (=2ν).

In TPEF, the energy of two less-energetic photons is used instead of a single high-energy photon to excite molecules from the ground state ($S_{0}$) to an excited electronic state ($S_{1}$). It is essential that the combined energy of the two photons is at least equal to the energy difference between $S_{0}$ and $S_{1}$ ($E_{min}$), and equivalent to the energy of a single photon, to excite the same molecules. Thus, typically, sources with wavelengths in the near-infrared (NIR) range are used for TPEF of molecules with high absorption coefficients in the ultra-violet (UV) range. Another precondition is the time gap between photon absorption. Both photons must interact with the molecule within a very brief time gap (in the order of 1 fs) [32,35]. Jablonski diagrams of one- and two-photon absorption are shown in Figure 2a.

Once the molecules are excited to $S_{1}$, they will undergo internal conversion (IC) followed by relaxation to $S_{0}$ via spontaneous emission of photons (fluorescence) or via nonradiative processes. The molecule’s quantum yield is commonly used as a metric for the probability of a molecule relaxing via radiative transitions. Both the fluorescence process and the molecule’s quantum yield are independent of excitation method used [35,36,37]. However, the molecule’s absorption cross-section, i.e., the capacity of the molecule to efficiently absorb light, is influenced by the excitation wavelength [37]. Nonetheless, for most molecules, two-photon light absorption spectra are equivalent to one-photon absorption spectra with wavelengths doubled [27].

TPEF has several advantages over linear excitation of the molecules in biological tissues. First, because NIR sources are used, tissue scattering is reduced, increasing the imaging depth that can be reached [34,36,38]. Second, the rate of nonlinear molecular excitation has a quadratic dependence on the excitation intensity, compared to the linear dependency of one-photon excitation. Thus, in TPEF, excitation is limited to the focal volume where the photon density is highest [36,37,38,39]. This gives rise to its inherent three-dimensional (3D) optical sectioning capability. Moreover, it also reduces tissue photodamage and photobleaching as they are limited to the focal volume [34,37,38]. In contrast, one-photon excitation occurs outside the focal plane. Tissue photodamage and photobleaching is widespread, and optical sectioning can only be achieved by blocking out-of-focus fluorescence with pinholes [37,39].

In biological applications, the characteristics of the fluorescence emission usually considered are its spectra, intensity, and lifetime. The fluorescence spectra allow the selection of the proper setup configuration, namely, the excitation wavelength and emission filters necessary for spectral separation of the fluorescence signals of interest. The fluorescence intensity indicates the number of photons emitted as a function of wavelength (emission spectrum). Fluorescence-emitted photons are less energetic than those that excited the molecules due to loss of energy during nonradiative relaxation processes (internal conversion) [36]. The fluorescence lifetime is the average time spent in the excited state and is computed from the inverse of the sum of the rate constants of all relaxation pathways [36]. Experimentally, it can be measured using fluorescence lifetime imaging microscopy (FLIM). For most fluorophores, the emission spectra and fluorescence lifetime are independent of the excitation type (one-photon or two-photon excitation).

#### 3.1.1. Fluorescence Lifetime Imaging

FLIM is an imaging modality that produces spatial and temporal resolved images of the sample fluorescence lifetime [40,41]. A complete review of FLIM principles can be found in Datta et al. [42]. Briefly, the measurement of the molecules’ fluorescence decay properties can be performed in time and frequency domain. With the latter, excitation is carried out using a light source with a high frequency modulation, typically assuming a sinusoidal profile. This will produce an equally modulated fluorescence signal, oscillating at the same frequency but with an attenuation in signal amplitude (demodulation) and a phase delay. The demodulation and phase are then used to retrieve the fluorescence lifetime [40,41,43,44].

**Figure 3:**
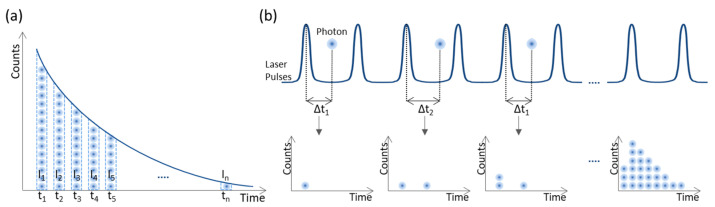
Schematic representation of time-gated (**a**) and time-correlated single photon counting (**b**) principles of operation.

In time domain, the fluorescence time response is directly measured as a function of time using ultra-fast detection systems. Molecules are excited using pulsed light sources [40,41]. Two main measurement techniques can be used: time-gated and time-correlated single photon counting (TCSPC). The principle of operation of both modalities is shown in Figure 3. Time-gated FLIM is a wide-field technique, i.e., all pixels within the image are acquired simultaneously. Pulsed laser light is used to excite the sample, and to generate trigger pulses, while an ultrafast gated intensified camera detects the fluorescence signals. The camera operates in a gated mode, i.e., the detection is switched between on and off states to create restricted time intervals, termed gates, during which fluorescence photons are acquired (Figure 3a). This results in a series of images (one for each gate) that can be used to reconstruct the fluorescence decay profile by plotting the photon counts versus the time of acquisition.

TCSPC is the most employed technique for fluorescence lifetime measurements due to its nearly ideal photon counting efficiency, high sensitivity, low SNR, and instrument response function (IRF) stability [45,46]. It measures the time delay between the excitation pulse and the arrival of individual photons. The laser source is used to generate trigger pulses. After photon detection, via photodetectors operating in single photon mode, the time difference between the trigger pulse and the pulse generated by photon detection is computed, converted into a digital signal, and stored. The fluorescence decay profile is inferred from the histogram of the arrival times of a high volume of photons (Figure 3b) [44,45,46]. From this principle it can be easily perceived that only one photon per excitation pulse can be timed. Therefore, having more than one photon arriving at the photodetector per excitation pulse would lead to an over-representation of short fluorescence lifetime photons (‘pile-up’ effect). However, TCSPC employs low-level, high-repetition rate signals with low intensity that drastically reduces the probability of more than one photon being generated per excitation pulse [44,45].

The fluorescence decay profile F(t) achieved using time-domain techniques typically takes the form of a multiexponential function as:

(1) $$F\left(t\right)=\overset{n}{\underset{i=1}{\sum }}a_{i}e^{\frac{-t}{\tau_{i}}}$$

where t is the time and $\tau_{i}$ is the fluorescence lifetime with relative contribution $a_{i}$. To retrieve the fluorescence lifetime and its contribution, curve fitting is usually performed. This is a time-consuming procedure and requires a priori knowledge such as the number of fluorescence lifetime components. It also requires a high number of photons for accurate fit of the data [47,48,49]. A minimum of 1000 photons per pixel is required for an accurate fit of a bi-exponential decay curve [49]. The quality of the fit is evaluated based on the weighted residuals curve and the reduced chi-squared ($\chi^{2}$). A good fit is characterized by residual values randomly distributed around zero and a $\chi^{2}$ close to one. Fit-free alternatives such as phasor plot are also used to retrieve the fluorescence lifetimes. In phasor analysis, the fluorescence decay in each pixel is converted into a vector and represented in a two-dimensional polar plot (phasor plot) [50]. Pixels with similar fluorescence lifetime will be clustered together, providing a visual overview of the fluorescence species present in the sample [50,51]. A complete overview of phasor analysis in fluorescence lifetime imaging has been recently published by Malacrida et al. [52]. Recent innovations in fluorescence lifetime data analysis are described in Section 5.3.

#### 3.1.2. Corneal Endogenous Sources of Contrast

TPEF and FLIM imaging based on endogenous sources of contrast offer the opportunity to retrieve information on the tissue non-invasively and label-free. Typically, biological tissues have a variety of endogenous fluorophores. The main sources of endogenous fluorescence in the corneal tissue are: (1) nicotinamide adenine dinucleotide and nicotinamide adenine dinucleotide phosphate (NAD(P)H) and (2) flavin mononucleotide and flavin adenine dinucleotide (flavins) present in the epithelial, endothelial cells, and keratocytes, and (3) collagen in the Bowman’s layer, stroma, and Descemet’s membrane. An overview of their fluorescence properties is shown in Table 1.

The autofluorescence of the metabolic co-factors NAD(P)H and flavins is commonly used to evaluate tissue metabolism. This is often referred to as optical metabolic imaging (OMI). The quantum yield of these molecules, and hence their autofluorescence, is highly influenced by their oxidation state. In the case of NAD(P)H, only the reduced form presents autofluorescence, while for flavins this is true only for the oxidized form. Thus, the ratio between the autofluorescence intensity of both metabolic co-factors can be used as a metric for the oxidation/reduction ratio within the cells and to infer the metabolic rate [53]. Nevertheless, the fluorescence intensity is also directly correlated with the fluorophore’s quantity within the cells. Thus, the fluorescence lifetime of NAD(P)H and flavins is typically preferred as an OMI tool. This fluorescence property is nearly independent of their concentration and highly influenced by the fluorophores microenvironment [41]. For this reason, free and protein-bound species of NAD(P)H and flavins can be easily discriminated. Because the oxidation and reduction processes are catalyzed by enzymes [54], the ratio between the free and protein-bound species of NAD(P)H and flavins can be used to infer the cells’ oxidation/reduction ratio [53,55,56].

Although NAD(D)H species separation could also be achieved by a blue-shift of the emission maxima when bound to proteins (Table 1), the proximity between the emission peaks substantially hinders this task [57]. The lifetime of protein-bound NAD(P)H and flavins differs at least 10-fold from their free counterparts. Free NAD(P)H has a fluorescence lifetime in the order of 0.4 ns [58]. When bound to proteins it drastically increases, reaching values between 1 and 6.5 ns, depending on the target protein [35,59,60]. For flavins the opposite behavior is observed. Free fluorescence lifetime values between 2 and 3 ns have been reported, with a decrease to 0.1 ns upon binding to proteins [35].

Both endogenous metabolic co-factors can be used independently to evaluate cells’ metabolic activity. Considering their two-photon cross-sections (Table 1), higher fluorescence emissions could be anticipated for flavins. However, the molecular concentration of NAD(P)H within the cornea is about 125 times higher than that of flavins [61,62], making the former the target of preference for corneal OMI.

**Table 1:** Fluorescence properties of endogenous fluorophores present in the cornea.

Endogenous Fluorophore	NAD(PH)		Flavins		Collagen
Two-photon excitation maximum (nm)	690–730		700–730 900		500–1700
Emission max (nm)	Free	Bound	525		380–520
	460	445			
Two-photon cross-section (GM)	0.02		0.08		-
Fluorescence lifetime (ns)	Free	Bound	Free	Bound	0.3 2.0–2.5
	0.4	1.0–6.5 *	0.1	2.0–3.0	
Reference Examples	[35,53,57,59,60]		[35,53]		[35,63,64,65,66]

* Depending on protein to which NAD(P)H binds. GM—Göppert–Mayer = $1\times 10^{-58}{\;m}^{4}s$.

Collagen is the most common intrinsic protein of the human body. This is also true for the cornea. Collagen presents intrinsic fluorescence and produces second-harmonic generation signals (see Section 3.2.1). The fluorescence excitation spectrum of collagen reaches its maximum around 340 nm [35,64]. However, two-photon excitation can be achieved over a broad range of wavelengths (Table 1) [63]. Fluorescence emission wavelength is dependent on the excitation wavelength. Higher excitation wavelengths lead to a red shift of the fluorescence emission [35,64,65,66]. Two lifetime components have been attributed to collagen (Table 1) [67,68]. These are known to be influenced by the level of crosslinking between the molecules [69,70]. An increase in collagen fluorescence lifetime has been associated with an increase in crosslinking level [69,70].

### 3.2. Second-Harmonic Generation

Like TPEF, SHG was first demonstrated shortly after the introduction of laser sources, by Franken et al. [71] in 1961. Its first application on biological samples was in 1971 by Fine and Hansen [72], with the first SHG images being obtained by Freund and Deutsch [73] in 1986. Since then, SHG has emerged as an important imaging modality with widespread applications in biological sciences.

SHG signals originate from the interaction of the material electric polarization with the optical radiation electric field [74,75]. The induced electric polarization (P) and the electric field (E) of the incident radiation are related by:

(2) $$P=\varepsilon_{0}\chi^{\left(1\right)}E+\varepsilon_{0}\chi^{\left(2\right)}E^{2}+\left(\ldots \right)$$

where $\varepsilon_{0}$ is the dielectric constant and $\chi^{\left(1\right)}$ and $\chi^{\left(2\right)}$ are the first- and second-order electric susceptibilities, respectively. Linear interactions with the molecules are described by the first term ($\varepsilon_{0}\chi^{\left(1\right)}E$), while the remaining terms correspond to nonlinear interactions. In molecules with noncentrosymmetry, the second-order electric susceptibility is different than zero ($\chi^{\left(2\right)}\neq 0$), giving rise to SHG [74,75,76,77,78]. In simple terms, the interaction of two photons of the same energy propagating at an angular frequency ω with a nonlinear material results in the generation of a single photon propagating at an angular frequency 2ω (Figure 2b).

To better understand the process of SHG, we must start with the correlation between the magnetic field and electric field of the optical wave (electromagnetic theory). From Maxwell’s equations [76,77,78]:

(3) $$\nabla \times E=-\frac{\partial B}{\partial t}$$

$$\nabla \times H=-\frac{\partial D}{\partial t}+J$$

∇·D=ρ

∇·B=0

where B is the magnetic field, H is the magnetic intensity, J is the free current density, and D is the electric displacement given by $D=\varepsilon_{0}E+P$, the wave equation for light propagating in a nonlinear medium can be obtained. For a non-magnetic medium ($B=\mu_{0}H$), without free charges (ρ=0), and without free currents (J=0), the wave equation takes the form [76,77]:

(4) $$-\nabla^{2}E+\frac{1}{c^{2}}\frac{\partial^{2}E}{\partial t^{2}}=-\mu_{0}\frac{\partial^{2}P}{\partial t^{2}}$$

where c=(μ0ε0)−12. Considering z as the wave propagation direction, and that in an anisotropic media ∇·E and ∇·P are zero, the wave equation can be simplified to:

(5) $$-\frac{\partial^{2}E}{\partial z^{2}}+\frac{1}{c^{2}}\frac{\partial^{2}E}{\partial t^{2}}=-\mu_{0}\frac{\partial^{2}P}{\partial t^{2}}$$

This equation must be valid for each frequency component of the electric field. In the case of SHG, the total electric field is:

(6) $$E\left(z,t\right)=\frac{1}{2}\left[E_{1}\left(z,t\right)+E_{2}\left(z,t\right)\right]$$

being the electric field of the components j=1, 2 given by:

(7) $$E_{j}\left(z,t\right)=E_{j}e^{i\left(\omega_{j}t-k_{j}t\right)}+c.c.$$

where c.c. is the complex conjugate, $\omega_{j}$ is the angular frequency, and $k_{j}$ is the propagation constant given by $k_{j}=\omega_{j}n_{j}/c$, where $n_{j}$ the refractive index of the medium at a frequency $\omega_{j}$ [76,77]. The total nonlinear polarization is:

(8) $$P\left(z,t\right)=\frac{1}{2}\left[P_{1}\left(z,t\right)+P_{2}\left(z,t\right)\right]$$

where $P_{1}\left(z,t\right)$ and $P_{2}\left(z,t\right)$ are:

(9) $$P_{j}\left(z,t\right)=P_{j}e^{i\left(\omega_{j}t-k_{j}t\right)}+c.c.,\;j=1,\;2$$

By replacing the complex forms of the electric field and electric polarization waves into equation (5), the coupled-wave differential equation that governs the SHG process can be derived:

(10) $$\frac{\partial E_{2}}{\partial z}=-\frac{i\omega d}{cn_{2}}E_{1}^{2}e^{i\Delta kz}$$

(11) $$\frac{\partial E_{1}}{\partial z}=-\frac{i\omega d}{cn_{1}}E_{2}E_{1}^{*}e^{-i\Delta kz}$$

where $d=\frac{1}{2}\chi^{\left(2\right)}$ is the nonlinear coefficient and $\Delta k=2k_{1}-k_{2}$ is the phase mismatch [76,77]. Since both electric fields ($E_{1}$ and $E_{2}$) are dependent on propagation distance z, Equations (10) and (11) must be solved simultaneously to retrieve the second-harmonic field ($E_{2}\left(z\right)$). Assuming that the fundamental field cannot be depleted ($E_{1}$ is constant), which is valid in the case of low energy conversion efficiencies, and the second-harmonic field being zero at the initial depth, equation (10) can be simplified to [76,77]:

(12) $$E_{2}\left(z\right)=-\frac{i\omega d}{cn_{2}}E_{1}^{2}e^{i\frac{\Delta kz}{2}}z\;sinc\left(\frac{\Delta kz}{2}\right)$$

where $sinc\left(\frac{\Delta kz}{2}\right)=sin\left(\frac{\Delta kz}{2}\right)/\frac{\Delta kz}{2}$. The intensity of second harmonic $I_{2}$ can then be obtained as:

(13) $$I_{2}\left(z\right)=\frac{1}{2}c\varepsilon_{0}n_{2}{\left|E_{2}\left(z\right)\right|}^{2}=\frac{2\omega^{2}d^{2}}{\varepsilon_{0}c^{3}n_{2}n_{1}^{2}}I_{1}^{2}sinc^{2}\left(\frac{\Delta kz}{2}\right)$$

Equation (13) shows that the second-harmonic intensity is proportional to the square of the fundamental intensity ($I_{1}$) and to the nonlinear coefficient (d). It also shows that the maximal intensity is achieved when Δk=0, implying that refractive index at frequency 2ω ($n_{2}$) is equal to the refractive index at frequency ω ($n_{1}$). This condition is typically denominated phase matching. For Δk=0, intensity increases quadratically with distance. This is dependent on the co-propagation of the excitation wave and the generated SHG signal, i.e., SHG propagates coherently and in forward direction coinciding with the excitation light direction. Without phase matching, it is proportional to $sin^{2}\left(\frac{\Delta kz}{2}\right)/\Delta k^{2}$ and maxima are reached at the coherence length $\left(L_{c}=\pi /\left|\Delta k\right|\right)$ [76,77].

Phase matching condition is not naturally present in most media. Being typically dispersive, $n_{1}$ and $n_{2}$ are different. Different solutions can be considered in this situation. When dealing with materials with birefringence properties, phase matching might be achieved using the ordinary and extraordinary refractive indexes [76,77,78]. Another alternative is to compensate for phase mismatch by creating a periodic dependence between the nonlinear coefficient d and the propagation depth z. This approach is commonly known as quasi-phase matching (QPM). The pooling period of alteration (Λ) has to be equal to twice the coherence length [76,77]. In this way, when mismatch is reached and the waves start to interfere destructively, leading to a decrease in the second-harmonic intensity, a change in the sign of the nonlinear coefficient allows it to increase continuously [77,78]. The nonlinear coefficient modulated periodically with a period Λ can be written as a Fourier series with m^th^ Fourier components as [77]:

(14) $$d\left(z\right)=d_{eff}\overset{\infty }{\underset{m=-\infty }{\sum }}G_{m}e^{iK_{m}z}$$

where $d_{eff}$ is the nonlinear coefficient of the homogenous material, $K_{m}$ is the grating wave vector given by $K_{m}=2\pi m/\Lambda$, and coefficient $G_{m}$ is given by $G_{m}=(2/m\pi )\;\sin(m\pi /2)$. The m^th^ component of the Fourier series is also referred to as the order of QPM. The highest efficiencies in QPM are achieved for lower order QPM (m=1).

Although QPM conditions increase the conversion efficiency, it is still lower than with perfect phase matching.

#### 3.2.1. Second-Harmonic Generation in Biological Tissues

**Figure 4:**
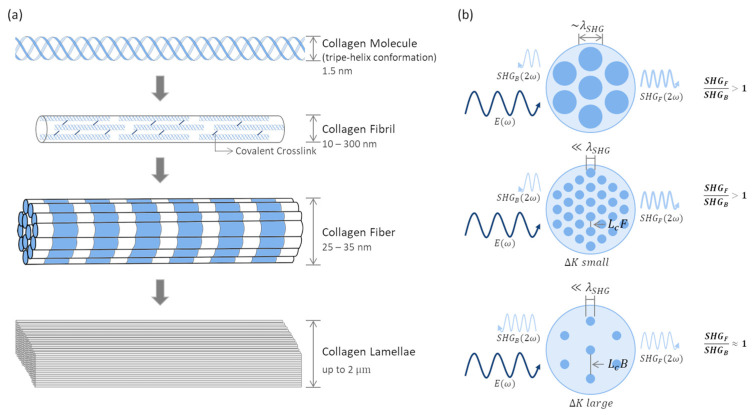
Corneal collagen hierarchic organization (**a**), and influence of fibril diameter and packing arrangement on the directionality and intensity of second-harmonic generation (SHG) signals (**b**). Arrows indicate the direction of signal propagation. E—energy; $SHG_{F}$ and $SHG_{B}$—forward- and backward-generated SHG; $L_{c}F$ and $L_{c}B$—coherence length of $SHG_{F}$ and $SHG_{B}$; $\lambda_{SHG}$—SHG wavelength; Δk—phase mismatch.

Collagen, due to its lack of center of symmetry, is an efficient SHG source in biological tissues [75,79,80,81,82]. Collagen has a fibrillar hierarchy structure and is often described as polycrystalline in nature. The fibrous proteins, composed of three peptide chains, are organized in tertiary triple-helix conformation. These can form covalent crosslinks in between to create fibrils that assemble into fibers (Figure 4a). In the corneal stroma, collagen fibers further self-assemble into lamellae [79,83].

In biological tissues, SHG is best described as a quasi-coherent process, i.e., SHG-generated signals propagate in the forward and in the backward direction. The latter component enables in vivo SHG imaging of biological tissues. Several sources have been proposed for the detected backward SHG signals. It has been associated with scattering of forward-generated signals [81,84,85] and backscattering of excitation photons that also generate SHG [81]. The most relevant contribution is backward-generated SHG photons. For this, the tissue inherent dispersion and randomness, the fibril diameter, and the packing density play important roles [86,87,88,89,90].

Mertz and Moreaux have demonstrated that sample inhomogeneities can provide axial momentum capable of altering SHG radiation patterns and, in specific conditions, generate backward SHG photons [86]. Considering collagen fibrils as the SHG-producing domains and collagen assembly, these can be approximated to a quasi-periodic lattice of scatterers. When the inter-fibrillary spacing and the coherent length of the generated SHG photons are of the same order, SHG signal intensity will be amplified by QPM interactions [87]. Backward-generated photons will build up when inter-fibrillary spacing matches the coherent length of backward SHG, associated with larger mismatch values, while the opposite occurs for forward SHG signals [87]. Based on these considerations, substantial backward SHG contributions can be expected when (1) the fibril diameter is smaller than the SHG wavelength [87,88,89], and (2) the packing arrangements have higher mismatch values and, consequently, shorter coherence lengths (Figure 4b) [87,89]. Fibril diameters in the order of the SHG wavelength, independent of their packing arrangement, and small fibril diameters in a densely packed arrangement, will generate mostly forward SHG (Figure 4b) [87,89]. It is important to notice that, in most cases, the forward-generated contribution is higher [87,88,91]. Maximal backward-generated SHG is achieved for fibril diameters in the order of 1/10 of the SHG wavelength, where it has an equal contribution to forward-generated signals (~50%) [87,88].

#### 3.2.2. Quantification of Tissue Organization

One of most widely used applications of SHG in biological tissues is the assessment and monitoring of collagen organization within the tissue. It can be used to differentiate between healthy and diseased skin [92] or to distinguish between healthy and tumorous tissue [93]. In the cornea, the high level of organization of collagen within the corneal stroma is essential to maintain its transparency and it is typically altered by external factors (e.g., pathologies and medical procedures). SHG is the ideal imaging modality to retrieve information on collagen orientation and evaluate the corneal collagen network. For this, quantitative metrics are paramount. Nevertheless, this is still an emerging area.

SHG photons are dependent on the polarization of the incident light. Thus, the intensity of the SHG signal depends on correlation between the orientation of the excitation beam and the collagen fibrils. Strong SHG signals will be obtained when the fibrils have the same orientation as the excitation light, whereas a perpendicular alignment will lead to weak SHG. Polarization-sensitive SHG imaging is perhaps the most straightforward way to quantify collagen organization and has already been employed to that effect [94,95,96,97,98,99]. However, acquisition of multiple images with different light polarizations drastically reduces the acquisition time and limits its application for in vivo measurements. Moreover, it is not suitable for imaging thick tissues since propagation through tissue can alter light polarization.

Circular polarized light is the type of illumination most used in SHG imaging of biological tissues, since it allows the collection of information from collagen fibers, independent of their orientation. Quantitative analysis of SHG must then be used to retrieve the embedded information on collagen orientation and overall level of organization. Several approaches have been proposed to retrieve this information and distinguish between tissues with different levels of organization. These include gray level co-occurrence matrix (GLCM) [92,93,100,101], structure tensor (ST) [102,103,104,105], and Fourier Transform (FT) analysis [101,106,107,108,109,110,111,112,113,114]. FT-based analysis is a popular and well-established approach. Using the fast FT algorithm, the image is decomposed into its frequency components, i.e., as a weighted combination of vertical and horizontal sinusoids. The most common metrics are the standard deviation of the angular distribution and the ratio between the short and long radii of an ellipse fitted distribution. GLCM is a statistical method often used in texture analysis. The idea behind it is to look at pairs of pixels, counting occurrences of all possible combinations in a particular direction and at a particular distance, creating the GLCM. From this matrix, one can extract several metrics such as correlation, energy, and homogeneity. Using structure tensor, collagen organization is obtained by computing a matrix of the partial derivatives along the x and y directions, containing information on the principal orientation and the degree of isotropy at every pixel.

All the metrics generated by these methods (GLCM, ST, and FT) have shown potential for the examination of collagen organization in the tissue. Nevertheless, they were employed in controlled settings aiming at the discrimination between sample types, with distinct collagen organization levels. They have not yet been objectively evaluated in images with known levels of organization, nor has their performance been compared.

## 4. Two-Photon Imaging Microscope Setup/Instrumentation

**Figure 5:**
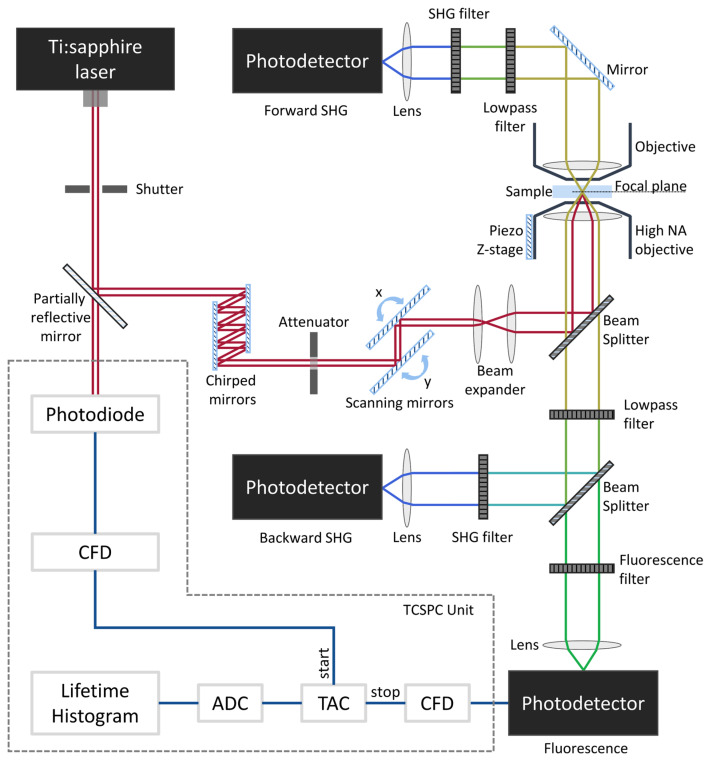
Schematic representation of the optical setup of a two-photon imaging microscope with multichannel detection combined with a time-correlated single photon unit for fluorescence lifetime imaging. CFD—constant fraction discriminator; TAC—time-to-amplitude converter; ADC—analog-to-digital converter.

TPI microscopes typically consist of a laser scanning platform with at least two detection channels to enable simultaneous detection of TPEF and SHG. These imaging modalities can be easily combined into a single system to provide complementary tissue information since they rely on distinct contrast mechanisms generated at distinct wavelengths that result from the same excitation. The typical setup of a TPI microscope with forward and backward detection channels combined with a TCSPC unit for FLIM imaging is shown in Figure 5.

Near-infrared (NIR) laser sources, with wavelengths ranging from 700 to 1000 nm, are typically used in TPI microscopes. Using NIR light, TPEF of tissue endogenous fluorophores with excitation spectra in UV range of the light spectrum is achieved while increasing tissue penetration depth. In addition, the reduction of the overlap between the excitation and fluorescence emission spectra allows for easy separation of the signals and reduction of fluorescence loss up to negligible values, when blocking the detection of the excitation source [37]. The choice of fundamental laser wavelength for SHG images is not as critical as for TPEF due to its non-resonant nature. Nevertheless, the acquisition of SHG signals generated at wavelengths below 380 nm is hindered by the limited glass optics transmission at these wavelengths [115]. Therefore, NIR light sources are typically selected, and the excitation wavelength is optimized for TPEF [85,116].

Key aspects of TPI microscopes are high spatial and temporal confinement. Two-photon absorption efficiency can be expressed as the number of photons absorbed ($n_{a}$) per fluorophore, per pulse:

(15) $$n_{a}\;\approx \;\frac{{\bar{P}}^{2}\delta_{2}}{\tau_{p}f_{p}^{2}}{\left(\frac{NA^{2}\pi }{hc\lambda }\right)}^{2},$$

where $\bar{P}$ is the laser’s average power, $\delta_{2}$ is the two-photon cross-section, $\tau_{p}$ and $f_{p}$ are the pulse duration and repetition rate, respectively, c is the speed of light, λ is the excitation wavelength, and NA is the numerical aperture of the objective lens [37,39]. The intensity of SHG signal is proportional to

(16) $$SHG\propto \;\frac{E_{p}^{2}\;NA^{2}}{2\pi \lambda \tau_{p}}{\left(\chi^{\left(2\right)}\right)}^{2}$$

where $E_{p}$ is the energy of laser pulse and $\chi^{\left(2\right)}$ is the second-order electric susceptibility [117].

From Equations (15) and (16), it can be perceived that the two-photon absorption efficiency and SHG signal intensity are proportional to the objective lens NA. Thus, the high special confinement required for TPI is achieved by using high NA objectives [36,37,38]. Since TPEF and SHG efficiencies are inversely proportional to the pulse duration of the laser ($\tau_{p}$), the temporal confinement of photons can only be accomplished through ultra-short laser pulses [37,117,118]. For this reason, solid-state mode-locked lasers, producing pulses in the order of fs at high repetition rates, have become the standard in TPI microscopes. Titanium sapphire (Ti:Sa) oscillators are typically used in biomedical applications due to their wide wavelength tuning range. To guarantee the arrival of ultra-short pulses at the sample, laser pulse dispersion (pulse broadening), caused by the optical elements of the beam path, can be pre-compensated using, for instance, chirped mirrors (Figure 5) [119,120,121].

As aforementioned, the excitation volume of two-photon processes is very small (order of 10^−15^ L). Therefore, scanning mirrors are used to raster scan the sample and acquire two-dimensional images. A vertical piezo stage may also be added to the instrument setup to obtain three-dimensional images.

Photon detection is accomplished by fast and sensitive photodetectors. Point photodetectors, namely photomultiplier tubes (PMTs), are the most employed. According to the signal’s characteristics, photons can be detected in reflection and/or transmission geometries. SHG photons can be detected in forward and backward configurations. Fluorescence signals are conventionally detected in reflection geometry. In addition to fluorescence intensity measurements, the photon’s arrival time may be recorded to obtain information in the fluorophore’s fluorescence lifetime. TCSPC is the most implemented FLIM method (Figure 5). This method relies on fast electronics to record the delay between the excitation and emission photons [44,45,46].

### Safety Considerations for In Vivo Two-Photon Imaging of the Cornea

MPI uses repetitive ultrashort pulses of NIR lasers. Hence, safety concerns regarding ocular tissue imaging exist. These pulses have high irradiance values that may produce optical breakdown of the irradiated tissue. The mechanisms involved in this process depend on the pulse duration. Femtosecond pulses can create a localized microplasma through multiphoton ionization caused by the high-intensity electrical field induced by the pulses. Free electrons in the microplasma start an avalanche effect, leading to plasma expansion and generation of a shock wave that moves at supersonic speeds and presents a high-pressure gradient at the wavefront. The tissue is split by the mechanical forces generated by the shock wave, a process named photodisruption. It can also be caused by cavitation and jet formation whenever optical breakdown occurs in soft tissues or fluids [122]. Photodisruption is used in surgeries like posterior capsulotomy of the lens, femtosecond-laser-assisted cataract surgery, and LASIK corneal refractive surgery.

The organization responsible for defining the maximum limits of exposure to laser radiation is the International Commission for Non-Ionizing Radiation (ICNIRP) [123,124]. These limits are the basis of laser safety standards like the IEC 60825-1 [125], adopted in Europe, or the ANSI Z136.1 [126], used in the USA. Any device for in vivo TPI of humans must comply with these standards. However, application of the rules concerning the calculation of the Maximum Permissible Exposures (MPEs) to TPI of the cornea is not straightforward. The worst-case analysis corresponds to a condition where the scanning of the sample by the laser beam is stopped, and the beam is focused on a single spot for the entire duration of the exposure. Although this condition simplifies the MPEs calculations, several difficulties remain: (1) MPEs are not available for pulse durations lower than 100 fs due to lack of data; (2) the standards assume the presence of protective mechanisms that are compromised during ophthalmological examination, namely blinking reflex and head movement after heating sensation of the cornea; (3) standards were developed for free illumination conditions, where a distant source illuminates an area larger than the eye pupil, while in TPI microscopy, light outputs from the microscope objective as a converging beam, enters the eye by a small aperture, and is focused on the cornea, diverging afterwards toward the lens and the retina.

A task group established by ICNIRP addressed the adjustment of the guidelines for safe exposure of the eye to optical radiation from ophthalmic light sources [127]. A statement was issued that recommends, for pulsed sources, an exposure limit of 1.8 t^−0.75^ $W/{\mathrm{cm}}^{2}$ for exposure durations less than 45 s, to protect against NIR hazard to the cornea and lens. This criterion was used by Ávila et al. in their paper reporting the first in vivo TPI microscopy images of the human cornea [128].

As the light emerging from the cornea will reach the retina, it is also necessary to comply with the maximum exposure levels for retinal irradiance. For TPI microscopy of the cornea with NIR radiation, hazard at the retina can be caused just by thermal mechanisms, since the use of high numerical aperture objectives to image the cornea will result in large retinal spots, preventing retinal irradiance values capable of producing mechanical effects. Due to thermal relaxation, retinal exposure limits for thermal interactions depend on the size of the illuminated area [123], which can be determined using optical design software with a standard human eye model and a model of the used objective [128].

ICNIRP guidelines and laser safety standards assume the protection given by the aversion response and by natural eye movements. This protection is compromised in ophthalmologic examination. To address this limitation, the task group established by ICNIRP adjusted the retinal exposure limits for pulsed illumination of large retinal areas, but only for spots with a diameter larger than 1.7 mm. The recommended limit is based on an ocular media transmittance of 0.9, and is given by 6 t^−0.25^ $W/{\mathrm{cm}}^{2}$, for exposures less than 10 s [127]. This limit, which is defined at the cornea, assumes that the pupil can constrict freely.

## 5. Advances in Two-Photon Imaging

### 5.1. Excitation Sources

The solid-state laser sources lasers typically used in TPI microscopes are costly, bulky, and require high maintenance. This hinders the full implementation of TPI into clinical practice. In recent years, a new generation of femtosecond fiber lasers has emerged that have matched and even surpassed the conventional solid-state lasers, due to their lower cost, smaller size, high performance, and reliability. In addition, fiber lasers do not require chillers or water cooling. The need for the complex alignment process of optical elements is also eliminated [129]. Thus, fiber lasers have opened the possibility to develop affordable and compact instruments for clinical applications.

The first fiber laser to achieve pulse parameters comparable to a conventional Ti:Sa laser was described by Kieu et al. [130]. This consisted of ytterbium (Yb)-doped gain fiber capable of generating 1060 nm pulses with widths of 80 fs at repetition rates of 70 MHz and average powers of 2.2 W [130]. Similar lasers have been used since to develop hand-held compact systems [131,132]. Liu et al. implemented a Yb-doped fiber laser generating 125 fs pulses at a central wavelength of 1060 nm with an average power of 1 W in a hand-held probe to obtain TPEF and SHG images of biological samples [131]. The feasibility of an all-fiber hand-held system for non-invasive SHG imaging of murine skin, as well as to monitor the penetration of fluorescence-labeled nanoparticles, has also been demonstrated [132].

Integration with endoscopic instruments is a natural application of fiber laser [129]. Recently, Akhoundi et al. reported on a multiphoton endoscope operating at 1700 nm for SHG and THG of unstained samples, and three-photon excitation of stained biological tissues [133]. The system consisted of a 40 MHz 1560 nm Erbium (Er)-doped fiber laser shifted to longer wavelengths using soliton self-frequency shift. The usage of longer excitation wavelengths is advantageous to achieve deeper penetration depths due to the reduction of water absorption and tissue scattering [133]. However, the excitation of endogenous fluorophores such as NAD(P)H is hindered, even when considering three-photon excitation [129,134].

Recently, a novel, compact, commercially available device employing a fiber laser was introduced for nonlinear, non-invasive imaging of the human skin (MPT*compact*, Jenlab GmbH). The laser generates 80 fs laser pulses at a fixed wavelength of 780 nm, which allows for label-free imaging of the skin endogenous fluorophores. This multimodal tomograph provides high-resolution and non-invasive TPEF combined with FLIM, SHG, and reflectance confocal images of the tissue. The MPT*compact* has already been used clinically to image healthy skin as well as pigmented skin lesions [135,136].

One drawback of fiber lasers is the fixed wavelength modality which, unlike solid-state lasers that are tunable over a range of wavelengths, limits the number of fluorophores that can be imaged with a single TPI device. Single-cavity multiple-wavelength fiber laser systems can overcome this limitation and enable simultaneous imaging of various fluorophores. Er-doped fiber lasers have great potential to achieve multiple excitation wavelengths. Wang et al. reported on an Er-doped fiber-laser-based system with simultaneous excitation at 775 nm, 864 nm, and 950 nm [137]. The different wavelengths were achieved through frequency doubling of the Er-doped fiber laser operating wavelength (1550 nm) and of the solitons generated by soliton self-frequency shift in a large mode-area fiber (1728 nm and 1900 nm) [137]. Chung et al. employed frequency doubling and self-phase modulation-enabled spectral selection to obtain 190 fs pulses at 750 nm and 47 fs pulses at 1250 nm, respectively, from an Er-doped fiber laser generating 290 fs pulses centered at 1550 nm [138]. This dual-wavelength femtosecond device was used for simultaneous TPEF of the ex vivo skin endogenous fluorophores, as well as SHG and third-harmonic generation (THG) of structural proteins [138]. An ultracompact, all-fiber, dual-wavelength, Er-doped fiber-laser microscope with excitation wavelengths of 790 nm and 1580 nm was also recently described [134]. Akhoundi et al. reported on an Er-doped mode-locked laser followed by a Yb-doped fiber amplifier to design a single-cavity laser emitting simultaneously at 1030 nm and 1700 nm [139].

### 5.2. Photodetectors

Conventional PMTs have been employed for several years in TPI instruments, particularly in TCSPC FLIM systems, due to their large active areas, low dark counts rates, photon-counting capability, and considerable adequate time response [140]. Nevertheless, the quantum efficiency of the conventional bi-alkali and multi-alkali cathodes is only about 20% to 25%, reaching its maximum between 400 and 500 nm. With the introduction of Gallium Arsenide Phosphide (GaAsP) cathodes, the sensitivity of PMTs increased up to 50% in a spectral range up to 700 nm [141,142]. Single-photon avalanche diodes (SPADs), also used in TPI setups, have higher quantum efficiencies (up to 80%) [141]. However, their small active areas (few 100 μm in diameter) hinder detection in non-descanned configuration [140,141,143,144].

Despite their merits, none of these photodetectors is optimized for FLIM measurements. Overall, they exhibit high after-pulse noise, increasing the background counts and leading to lifetime inaccuracies [140,141,142]. Moreover, the time response of these detectors, given by the full-width half-maximum (FWHM) of the instrument response function (IRF), is in the order of 300 ps, which is insufficient for high-end FLIM applications [142]. These limitations were overcome with the introduction of hybrid detectors (HPD). These detectors combine the vacuum tube of a PMT with photodiode semiconductor technology. HPDs have high quantum efficiencies and active areas comparable to those of a conventional PMT (~5 mm diameter). They are also free of after-pulsing, which greatly increases lifetime accuracy [140,141,142]. Additionally, temporal resolutions of 20 ps can be reached [145].

The possibility to further increase temporal resolution in TCPSC measurements by using superconducting nanowire single-photon detectors (SSPDs) has been recently reported [146]. SSPDs can achieve temporal resolutions of about 2.7 ps for visible wavelengths, the shortest ever reported [146]. Like HPDs, in SSPDs after-pulse background is absent. The major drawbacks of these detectors are their small active area (~10 μm) and their low operating temperature (≤4 K) [141,142].

### 5.3. Analysis Methods

In recent years, advances in computing power and artificial intelligence, together with the emergence of deep learning, have led to a revolution in how data is processed. The use of machine learning, and more specifically deep learning, is now widespread in many fields. Naturally, the medical sciences have followed suit. These novel approaches have already been used efficiently, achieving impressive performance in tasks such as disease diagnosis, detection of lesions, or segmentation of relevant structures, within a wealth of medical image fields and modalities.

At the base of this revolution are neural networks, inspired by a simplified model of the brain, composed of interconnected nodes or neurons that can pass information between them like synapses. These neurons are organized into layers. The simplest neural network is composed of just an input and an output layer. Naturally such a simple structure typically fails to model complex interactions. The solution is additional layers in between input and output, called hidden layers. The ‘deep’ in deep learning is in reference to the number of layers in the network.

As discussed, fluorescence lifetime decay curves analysis is time-consuming. This is exponentially more concerning as advances in instrumentation make data acquisition faster. Systems with high throughput acquisitions are becoming the norm. Nevertheless, fit-based FLIM approaches are still the standard. These are iterative, slow processes that require experienced user supervision and manipulation in the form of initial conditions setup or even manual corrections. The use of deep learning to accomplish a fit-free FLIM promises to eliminate manual interventions and provide a significant speed-up, with the goal of real-time FLIM. In 2016, Wu et al. showed that an artificial neural network was able to accurately estimate fluorescence lifetimes faster than a least square fit-based alternative and without the need of initial conditions setup [147]. Since then, other approaches based on convolutional neural networks (CNNs) have been proposed. CNNs are the gold-standard neural networks architecture for image analysis. Characterized by its main layer type, the convolutional layer, the network uses scanning kernels to convolve the input, enhancing efficiency and making it possible to learn more complex feature representations. Fit-free analysis of decay curves by CNNs has been shown for both mono- and multi-exponential decays, without the need for preconditioning, matching or even out-performing fit-based alternatives, especially under low-photon-count conditions [148,149,150].

Beyond fit-free FLIM, deep learning can also have an important role in the analysis of obtained images. Guimarães et al. applied a CNN-based approach to detect living cells and diagnose atopic dermatitis from two-photon images (TPEF and FLIM) of the human skin, obtaining an accuracy exceeding 97% [151]. Saliency maps to improve on the explainability of these deep learning models were used [151]. One of the main disadvantages of these non-linear models is their ‘black box’ nature, i.e., it is impossible to know exactly why a given decision was made by the algorithm. This naturally hinders their wide application and acceptance, especially in the medical field, where ethical and regulatory concerns exist. In the future, the implementation of further strategies to enhance the explainability of these methods is mandatory.

As with FLIM, SHG images have also been used together with deep learning to show the potential of TPI. In 2017, Liang et al. used CNNs to predict tissue elastic mechanical properties, identifying the overall tissue stiffness with 84% accuracy and predicting the nonlinear anisotropic stress–strain curves with 0.021 and 0.031 average errors [152]. In 2018, Judd et al. applied CNNs in a pilot study to distinguish between benign renal oncocytoma and malignant chromophobe renal cell carcinoma, reaching an accuracy of 68.7% [153]. Mirsanaye et al. proposed for the first time that polarized SHG imaging of the collagenous extracellular matrix combined with CNNs could be used as histopathological biomarker of cancer [154]. Other applications of deep learning with SHG images include the quantification of scar collagen texture and prediction of scar development [155], and automated collagen segmentation [156].

## 6. Two-Photon Imaging of the Cornea

**Figure 6:**
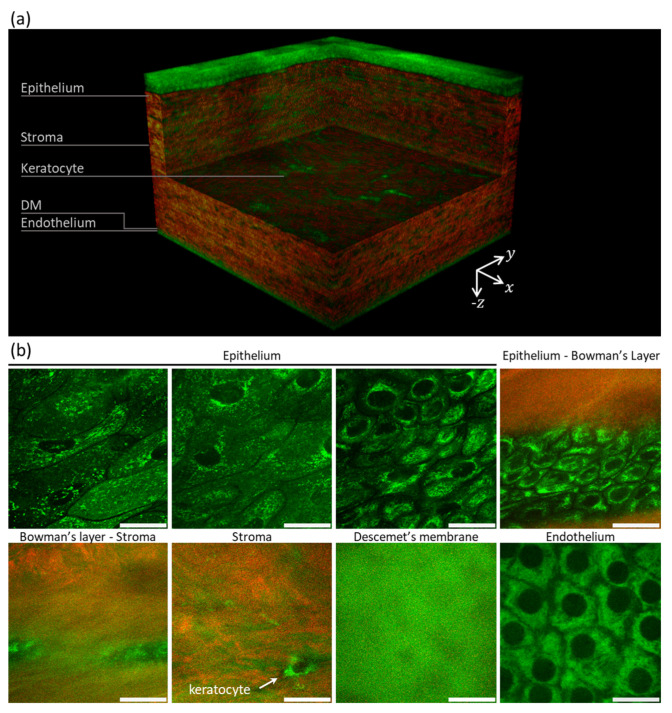
3D composite representation of the porcine cornea reconstructed from autofluorescence (green) and second-harmonic generation (red) images of the tissue (**a**), and depth-wise en-face images of the human cornea autofluorescence (green) and second-harmonic generation (red) (**b**). Images were acquired with the multiphoton tomograph MPT*flex* (JenLab, GmbH) using an excitation wavelength of 760 nm. Scale bars = 20 μm.

Using TPI microscopy, the different layers and sublayers of the cornea can be evaluated. The distribution of endogenous fluorophores within the cells enables their morphological characterization and discrimination into superficial, wing, basal, and endothelial cells. SHG signals are generated from collagen fibers in the Bowman’s layer and stroma. In the latter, AF signal from keratocytes is also detected. Being mainly composed of nonfibrillar collagen (collagen type IV), the Descemet’s membrane shows AF but no SHG signals (Figure 6).

### 6.1. Two-Photon Imaging for Non-Invasive Corneal Evaluation

The first TPEF images of the cornea were acquired by Piston et al. in 1995 [157]. In this work, the authors employed a 705 nm ultrafast dye laser, generating 150 fs pulses, for two-photon excitation of the NAD(P)H AF [157]. Layer discrimination based on cell morphology was possible. Moreover, AF intensity changes related with metabolic changes were observed [157]. The feasibility of using the metabolic co-factors AF to assess corneal cells’ metabolic state had already been extensively demonstrated through redox fluorometry [158,159,160,161,162,163]. Nevertheless, Piston et al. were the first to report two-dimensional images of corneal cells metabolism based on TPEF. Two years prior, the first corneal images based on one-photon excitation fluorescence had been reported by Masters et al. [164].

Soon after the demonstration of SHG, its feasibility to record collagen-generated SHG was also demonstrated [72]. SHG images of ex vivo corneas were first recorded in 2002, by König et al. and Yeh et al. to characterize porcine and rabbit tissue, respectively [165,166]. Ever since, several groups have employed TPEF combined with SHG to assess the morphology of animal corneal tissue ex vivo [167,168,169,170] and in vivo [94,171,172,173,174,175]. In 2010, Aptel et al. characterized human corneal buttons morphology, over its entire thickness, based on a multimodal approach combining TPEF, SHG, and THG [176]. Recently, a TPI microscope was optimized for the first in vivo evaluation of corneal morphology [128]. The device employed a Ti:Sa femtosecond laser, generating 395 fs pulses with a central wavelength of 800 nm and repetition rate of 76 MHz for tissue excitation. A long-working-distance, non-immersion focusing objective was used to avoid eye-contact [128]. The authors reported, for the first time, in vivo SHG images of the corneal stroma and sclera of healthy volunteers as well as trabecular meshwork SHG and TPEF images [128].

Corneal metabolic assessment based on two-photon excited FLIM was first demonstrated by König and coworkers [56]. In their work, the authors characterized mouse, rabbit, porcine, and human corneas based on the metabolic co-factor NAD(P)H AF lifetime. Additionally, alterations to human corneal epithelial cells’ metabolism with storage, based on NAD(P)H AF lifetime, were evaluated [56]. Recently, Batista et al. monitored the status of human corneal buttons with storage time, based on their morphology, metabolism, and structural organization, using a TPI microscope [101]. By combining TPEF, FLIM, and SHG, the authors observed (i) corneal endothelial cells’ morphological changes and the characteristic cell loss; (ii) an increase in NAD(P)H free to protein-bound ratio of epithelial and endothelial cells, consistent with a decrease in these cells’ metabolism; (iii) an increase in keratocytes’ metabolic activity due to the activation of these cells; and (iv) a decrease in the stromal collagen-fibers organization, detected using FT analysis and GLCM, indicative of early changes in corneal transparency [101].

### 6.2. Clinical Applications of Two-Photon Imaging

One of the most important ophthalmological applications of TPI is in the diagnosis of corneal pathology and treatment follow-up. The potential of TPI to diagnose and monitor different corneal diseases was already demonstrated. An overview of clinical TPI applications is shown in Table 2.

Tan et al. showed that TPEF and SHG can be used to detect corneal morphological alterations induced by bacteria and fungi (*Serratia marscecens*, *Alternaria*, *and Acanthamoeba-Pseudomonas aeruginosa*) [177]. Moreover, pathogen specimens could be identified and differentiated without additional histological processing [177]. Along with changes to cell morphology, a decrease in cell metabolic activity has also been reported in *Acanthamoeba* infected tissue [114]. Information on the infiltration mechanisms of *Pseudomonas aeruginosa* in ex vivo and in vivo rabbit corneas has also been revealed using TPI [174].

SHG and TPEF imaging have been shown to be a relevant tool to diagnose corneal edema, revealing alterations to collagen architecture and keratocytes activity [105,176,178]. Steven et al. employed TPEF to investigate the transmigration of immune cells in animal models of corneal neovascularization [175]. Metabolic changes during corneal wound healing, based on NAD(P)H AF lifetime, were also monitored by Gehlsen and coworkers [179]. Recently, SHG imaging combined with ST for quantitative evaluation of collagen organization was employed to monitor corneal stroma architecture following chemically induced burns [103]. According to the authors, normal levels of collagen organization can be reached 6 months after burning [103].

Due to its pathophysiology, the possibility to improve keratoconus diagnosis based on TPI has been evaluated by several groups. Keratoconus is a progressive ectatic disorder characterized by stromal thinning and corneal protrusion into a conical shape, leading to irregular astigmatism and vision loss [195]. Thus, SHG is the ideal tool to assess morphological and structural changes induced by keratoconus and has already been efficiently used for that effect [99,105,114,180,181,182,183]. Changes to the stroma collagen lamellae distribution [180], orientation [181,182], interweaving [183], and overall organization [99,105,114] have been observed in disease-affected corneas. Quantitative changes have been assessed using polarization-SHG [99], ST [105], and FFT [114,182]. Additionally, changes to the corneal epithelium and stroma AF lifetime due to keratoconus were also reported, adding new complementary information to SHG data [114].

In the past years, TPI has also been employed both to improve the methodology of keratoconus treatment and to monitor patient evolution after treatment. Corneal collagen crosslinking (CXL), first introduced in 2003, strengthens the tissue through the formation of new covalent bounds between collagen molecules and fibrils [196]. This is achieved by the photodynamic reaction between ultraviolet A (UVA) light and riboflavin [196]. Being a recent clinical procedure, efforts to improve treatment efficiency and decrease patient discomfort have been carried out. In this review, we focus on those involving TPI applications. Seiler et al. has demonstrated that riboflavin levels at the endothelium were substantially below the theoretically calculated ones, showing that higher radiant exposures could be applied during CXL, improving treatment efficiency [197]. Moreover, these results also indicate that CXL could be performed in thinner corneas, increasing the pool of eligible patients [197]. Riboflavin two-photon excited fluorescence was used to determine its concentration [197]. The possibility to increase transepithelial riboflavin penetration in rabbits corneas by creating femtosecond laser-induced microchannels was recently demonstrated [198].

Two-photon activation of riboflavin, thus increasing the CXL efficient volume and its specificity, is also a topic of interest and has been a subject of research over the years. First demonstrated in tissue-mimicking samples [199,200], corneal non-linear CXL, i.e., CXL using two-photon excitation, was later demonstrated in 2016 [190]. Femtosecond laser was focused on de-epithelized riboflavin-embedded bovine corneas using a high NA objective to achieve highly localized treatment [190]. However, long treatment times (1 h per mm^2^) were required to irradiate large volumes. To decrease irradiation times, Bradford et al. [191,192] proposed and demonstrated two-photon excited CXL in rabbit corneas using low NA objectives. By enlarging the focal area, ~1.4 mm^3^ could be crosslinked in about 11 min. However, this was reliant on reduction in treatment specificity and an increase in the mean laser power, well above hazardous values. Recently, the two-photon excited CXL in human tissue was also demonstrated [193,194].

A successful CXL treatment will lead to changes in the structural organization of collagen fibers within the stroma. Therefore, TPI shows great potential for patient follow-up. CXL-induced changes to collagen fibers organization were already demonstrated by several groups, using SHG imaging [108,184,185,186,187]. Based on forward-detected SHG images, an increase in collagen fiber waviness following CXL was observed after FT analysis [108]. Mercatelli et al. applied the ratio between forward- and backward-detected SHG signals to characterize the structural organization of collagen in healthy, keratoconus, and CXL-treated keratoconus samples [186]. As anticipated, the authors found a decrease in stroma structural organization in keratoconus samples with re-arrangement of collagen fibers to values comparable to healthy corneas after treatment [186]. The major disadvantage of this method is its impossibility for in vivo implementation.

**Figure 7:**
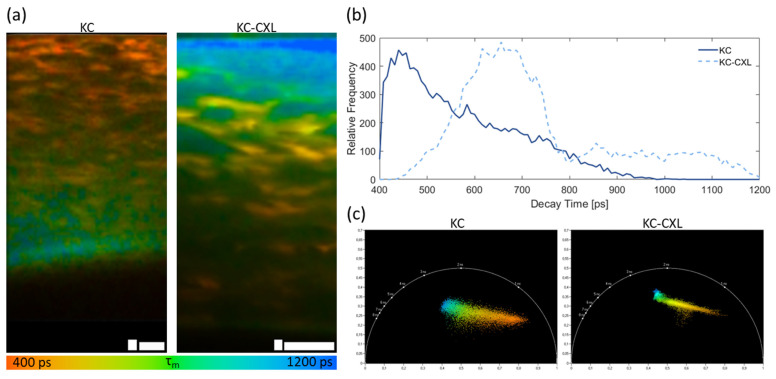
Cross-sectional autofluorescence (AF) lifetime images color-coded for mean AF lifetime (as indicated in the color bar) of the keratoconus (KC) and keratoconus crosslinked (KC-CXL) corneas 24 h after treatment (**a**), corresponding mean AF lifetime decay distributions (**b**) and phasor plots (**c**). Images were acquired with the multiphoton tomograph MPTflex (JenLab, GmbH) using an excitation wavelength of 760 nm. Fluorescence lifetime data analysis was performed using SPCImage vs 6.2 (Becker & Hickl GmbH, Berlin, Germany). Scale bars = 20 μm.

Although SHG is the undeniable choice to evaluate collagen fiber organization, CXL-induced changes to the corneal tissue are also detected through TPEF and FLIM imaging. Steven et al. verified that AF intensity and lifetime of the rabbit’s corneal stroma is increased two weeks following CXL [188]. More recently, Batista et al. showed that AF intensity and lifetime of the stroma of human corneal buttons is altered after CXL, in connection with the increase in covalent bonding between collagen [189]. These effects are noticeable as soon as 2 h following treatment [189]. A preliminary study also showed an increase in the tissue collagen AF lifetime, combined with keratocytes depletion in CXL-treated keratoconus samples (Figure 7) [7].

Along with corneal diseases, TPI can also be an important instrument for the diagnosis of systemic diseases through corneal imaging. Morphological and structural changes to the corneal tissue, namely, an abnormal formation of collagenous structures in the Descemet’s membrane, have been reported in corneas of diabetes type 2 patients [201,202]. Moreover, TPI can be used to quantify the accumulation of advanced glycation end products (AGEs) [203]. These components are believed to be highly correlated with the development and progression of several systemic diseases, such as diabetes mellitus and Alzheimer’s disease [204,205].

## 7. Conclusions and Future Directions

TPI microscopy has the potential to overcome the limitations of current corneal clinical imaging modalities. It provides structural imaging in all corneal layers, with performance comparable to corneal confocal microscopy, and it adds the possibility of assessing the metabolism of corneal cells and of imaging, through SHG, the organization of stromal collagen fibers.

These features support the promise of the relevant impact of a future TPI clinical device for combined structural and functional examination of the cornea. Imaging the structural organization of stromal collagen fibers will contribute to a better understanding of the pathophysiology of diseases like keratoconus or myopia. The evaluation of lamellae organization and fibril orientation within lamellae by SHG may also provide earlier diagnosis of keratoconus, as well as better follow-up and assessment of therapy. Regarding myopia, there is interest in evaluating collagen ultrastructure since it seems to correlate with tissue biomechanical alterations. However, although corneal stromal organization may be related to myopia pathogenesis, the tissue most relevant to this pathology is the posterior sclera. Structural two-photon imaging may also be useful to assess processes of corneal wound healing.

The evaluation of cells’ metabolism will contribute to reaching a long-held objective in ophthalmology, giving the clinician the advantage of diagnosing corneal cells’ dysfunction prior to its pathologic expression. Corneal metabolism can be affected by local or systemic disorders. Relevant local disorders include Acanthamoeba Keratitis, Fuchs’ dystrophy, and contact-lens-induced metabolic changes. Systemic metabolic disorders produce corneal manifestations, namely, alterations of its transparency, that are termed metabolic keratopathy. The most prevalent systemic metabolic disorder is diabetes mellitus. Other conditions include disorders of lysosomal storage (mucopolysaccharidoses, lipidoses, cystinosis and others), disorders of lipid metabolism, and disorders of amino acid, nucleic acid, and protein metabolism. For all these pathologies, it is expectable that alterations in corneal cells metabolism will occur before structural alterations, justifying the potential of TPI microscopy for early diagnosis of corneal dysfunction.

Clinical TPI microscopy may also significantly impact the procedure and the evaluation of the outcome of CXL. While currently this outcome is typically evaluated several weeks after treatment, TPI seems able to provide a valid assessment on the same day of the treatment, allowing an immediate re-intervention in case of failure. Moreover, the prospect of using two-photon photoactivation of riboflavin for keratoconus treatment brings forward the possibility of having a single device for treatment and immediate evaluation.

Although a very significant breakthrough occurred with the recent publication of Ávila et al. [128], reporting the first images of the in vivo human cornea, clinical application of corneal TPI is still distant, and several challenges remain: (1) safety and stability issues were properly addressed and allowed using in vivo SHG imaging, but thus far in vivo TPI of corneal cells, based on NAD(P)H) and/or flavins, has not been demonstrated; (2) TPI instrumentation is very expensive and complex, being difficult to use in a clinical environment; (3) SHG is the ideal imaging modality to evaluate the corneal collagen network, but it is necessary to develop reliable quantitative metrics for retrieving information from backward-direction SHG images.

Concerning instrumentation, it is important to note that the prices of femtosecond lasers have been steadily decreasing as a new generation of fiber lasers has emerged, offering smaller size, better reliability, and much improved usability. The drawback of fixed wavelength emission may be adequately overcome by single-cavity, multiple-wavelength fiber laser systems and by future developments concerning Er-doped fiber lasers. Likewise, the introduction of hybrid detectors represented a great improvement in timing resolution and constituted a significant step toward a clinical instrument. The recent introduction of a commercial compact device, based on a fiber laser, for human skin imaging, signals the current trends of TPI instrumentation for use in clinical environments.

Improvements in SHG and FLIM data analysis are also required. Although several metrics were proposed to assess the level of collagen organization, data analysis of corneal SHG images is still mostly qualitative and existing methods never have been objectively evaluated or compared. Studies comparing the performance of different metrics in controlled conditions, like those provided by using computer-generated SHG images, are required to progress to clinical applications.

It is also important to develop new FLIM data analysis methods to comply with usability constraints posed by operation in clinical settings. Current fit-based techniques are time-consuming and require experienced user supervision. Deep-learning-based methods hold great promise and will certainly change the state-of-art in FLIM data analysis. Approaches based on CNNs can accomplish fit-free FLIM, solving the constraint of user supervision. Deep learning methods may also greatly improve the image analysis process, but it is necessary to enhance their explainability to be widely accepted for clinical use.

In conclusion, several key developments must be accomplished for translating corneal TPI to clinical environments. However, the potential value of the additional information provided by TPI, compared to current clinical corneal imaging methods, is a substantial driving force for fostering those developments. In the future, corneal TPI will certainly be a reality in ophthalmology departments, improving disease diagnosis and therapeutical evaluations.

## Figures and Tables

**Table 2:** Overview of two-photon imaging clinical applications.

Condition	Key Findings	Reference Examples
Corneal Infections	TPEF and SHG showed the structure of epithelial cells. FLIM showed a decrease in epithelial cells metabolism. TPEF enabled discrimination between different pathogens. TPEF used for in vivo tracking of pathogen infiltration.	[114,174,177]
Corneal Edema	SHG showed a decrease in corneal stroma structural organization based on qualitative and ST analysis. TPEF showed keratocytes activation.	[105,176,178]
Corneal Neovascularization	TPEF used to observe in real-time the transmigration of immune cells into lymphatic vessels.	[175]
Wound Healing	FLIM showed an increase in metabolic activity of epithelial cells after damage. SHG showed a loss of collagen organization following induced damage, with progressive re-organization up to full recovery (six months). Changes were quantitively assessed using ST analysis.	[103,179]
Keratoconus	TPEF detected structural alterations to epithelial cells. FLIM showed a decrease in corneal epithelial cells metabolism. SHG showed changes to collagen structural organizations. These have been evaluated qualitatively and quantitatively using ST, FT analysis, and using polarization SHG measurements.	[99,105,114,180,181,182,183]
CXL Evaluation	TPEF and FLIM showed an increase in stroma AF intensity and lifetime, consistent with an increase in the amount of crosslinking, as soon as 2 h after treatment. SHG showed changes in collagen structural organization after treatment, quantifiable using ST, FT, and the ratio between forward and backward SHG.	[108,184,185,186,187,188,189]
Two-photon induced CXL	Two-photon photoactivation of riboflavin can efficiently increase corneal stiffness.	[190,191,192,193,194]

TPEF—two-photon excited fluorescence; SHG—second-harmonic generation; FLIM—fluorescence lifetime imaging microscopy; ST—structure tensor; FT—Fourier Transform.

## Data Availability

Not applicable.
